# Redispersible polymer powder modified cementitious tile adhesive as an alternative to ordinary cement-sand grout

**DOI:** 10.1016/j.heliyon.2021.e08411

**Published:** 2021-11-17

**Authors:** Mashrafi Bin Mobarak, Md. Sahadat Hossain, Monika Mahmud, Samina Ahmed

**Affiliations:** a Glass Research Division, Institute of Glass & Ceramic Research and Testing, Bangladesh Council of Scientific and Industrial Research (BCSIR), Dhaka, 1205, Bangladesh; b BCSIR Laboratories Dhaka, Bangladesh Council of Scientific and Industrial Research (BCSIR), Dhaka, 1205, Bangladesh

**Keywords:** Building material, Ceramic tiles, Tile adhesive, Redispersible polymer, Cementitious material

## Abstract

With the advent of technologies on modern structural and building materials, adaptation of such technological features has been the pivotal concern of researchers. Ceramic tiles attained a distinctive focus due to its decorative feature for both indoor and outdoor conditions and also for their ease of implementation. The purpose of exploiting ceramic tiles is not only to impart structural strength but also the aesthetical characteristics that seem to matter a great deal. When it comes to the adhesion of these tiles onto the substrate wall, traditional method exerts the use of sand-cement grout. This faces some drawbacks including poor water retention property, hard and brittleness of the surface, much higher drying time, no flexibility, higher thickness of the paste and so on. These difficulties can be overcome by the addition of redispersible polymer powder (RPP) along with other cementitious constituents. The blended polymers interact with cement components to improve the physical and mechanical properties such as increased adhesion strength, reduced shrinkage and lower water absorption. This review article made an effort to provide the generalized idea about the cementitious tile adhesive (CTA) and its components. Focus was made onto the commercially available RPP and formulation of CTA with the inclusion of RPP. Critical analysis of the repercussions of RPP fortification was also carried out based on different researcher's findings.

## 1 Introduction

Being the oldest form of decorative art, ceramic tiles, from its inception to evolution through ages, is laden with history, from ancient Egyptian artifacts to modern household decoration. Their enthralling beauty and structural permanence made them treasured for centuries. The oldest evidence of ceramic tiles usage was dated from 4700BC in Egypt; subsequently, Romans and Greeks also started decoration with ceramic tiles. During the Islamic period, decorative ceramic tiles were frequent in mosques. Decorative tiles got admiration at Europe during the middle age and it was the industrial revolution of Britain which made the mass production possible as well as the affordability for the middle class people [1].

External cladding of tiles, also known as tiling system [2] has become the most picked construction choices not only for its aesthetic purposes but also for the patronage of wall from aggressive environmental conditions [3]. Ceramic tiles are the most adopted tiles around the world due to its versatility [2] and affordability [4]. The ceramic tiling system comprises of three interactive layers; the substrate layer, the tile layer and most importantly the adhesive layer [3, 5]. Adhesives refer to the substance, which has the ability of holding minimum two surfaces substantially in an indissoluble way. Adhesives that are used in structural purposes must possess high shear strength and a very strong resistance from aggressive environment [6].

Traditionally for the external cladding of ceramic tiles, a cheaper form of adhesive which is nothing but a mixture of cement and sand, is being used widely and much popular in Indian subcontinent. Traditional grout preparation involves mixing of ordinary portland cement and sand with water and the application of this grout is done by following the thick bed method, where the grout bed occupies 10–25mm of thickness from the base to the adherent [7]. This is a very time consuming process and requires much effort. Application of a polymer modified thin bed tile adhesive is a possible solution to all the problems raised by traditional cement-sand grout [8].

Polymer based adhesives are introduced to cement as a modifier for the betterment of adhesion, strength, waterproofing, durability, flexibility and deformity. Polymer modification of cement grout or paste for tiling and other purposes is not a contemporary conception; rather it had been performed since 1923. In 1924, the first patent regarding polymer modification in cement mortar was issued. Since then, polymer modified cement grout got the implementation in construction arenas for providing a decent performance compared to that of the ordinary cement-sand grout and eventually got the popularity [9, 10].

**Figure 1:**
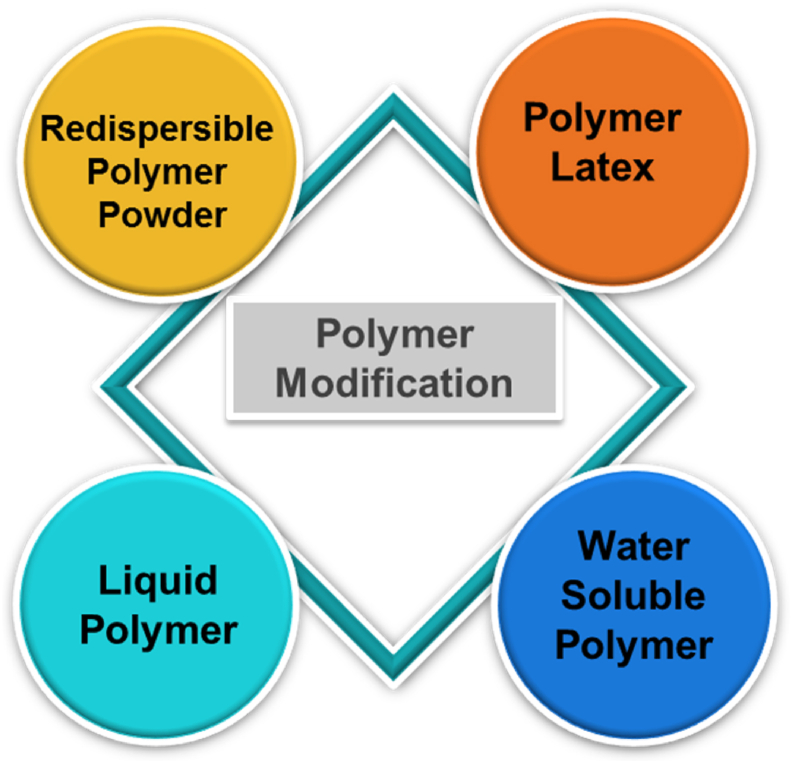
Different types of polymer modification.

The basic principle of polymer modification involves the mixing of polymer or monomer in powdered or liquid form along with cement and other admixtures followed by curing. In case of monomers being used, in-situ polymerization of that monomer is required. The polymers or monomers with which polymer modification can be done are of four major types (Figure 1), i.e., RPP, polymer latex, water soluble polymer and liquid polymer [9].

RPPs are simply the spray-dried powders which when mixed with water, produce stable dispersion just like the original dispersion and this is governed by the fact that it redisperse only once [11]. Originally, RPPs are synthesized from latex dispersions and this involves a two-step synthesis process. The first step includes preparation of polymer latexes via emulsion polymerization and the second step being the spray drying of this emulsion into fine powder. Prior to the spray drying, various admixtures are added to the formulation such as anti-foaming agents, accelerators, water retention agents etc. for improving the performance of the polymer. To prevent the cake formation of the polymer powder during storage, anti-blocking aids such as clay, silica and CaCO_3_ are added before or after the spray drying [9].

Polymer latexes or polymer dispersions are synthesized just like RPP by emulsion polymerization. Polymer latexes are characterized by the fact that they are of very small particles, typically possesses 0.05–5μm in diameter. Common examples of synthetic latexes include vinyl acetate, homo- and co-polymer latexes, acrylic copolymer latex, styrene butadiene copolymer latex etc. Due to chloride ion liberation and unsatisfactory resistance, polyvinyl acetate and poly (vinylidene chloride-vinyl chloride) latexes are not advised. In case of natural rubber and epoxy latexes, they are not compatible to be synthesized by emulsion polymerization, rather they are acquired naturally by tapping from the rubber trees, which is then concentrated and finally a solid mass is obtained. Cellulose derivatives, polyvinyl alcohol, polyacrylamide etc. are examples of water soluble polymer which are mixed with cement in powdered form with the expectation of preventing the dry out occurrence and to improve the serviceableness. This is due to the increasing viscosity of water by dissolving the polymer and the sealing property is exerted by the formation of a thin film. Overall, the modification with water soluble polymer doesn't contribute substantially to the strength of the system. Liquid polymers find the least of utilization due to its handling and storage difficulties. They are mainly epoxy and unsaturated polyester resins which are applied along with hardener or catalyst and accelerators [12].

CTA modified with RPP has got the special consideration because of its beneficial aspects. This whole concept is justified by the fact that, the hydration of cement and formation of polymer film occurs accordingly which begets a network structure of monolithic matrix phase through which the hydrated cement phase and polymer phase interpenetrate. Modification with RPP is akin to latex modification but with the advantage of the redispersion property [13]. Some of the features delivered by RPP are improved workability, improved adhesion, increased flexural strength, increased plasticity, improved abrasion resistance, improved water retention, reduced water absorption, increased viscosity and cohesion etc. Inclusion of RPP in the cement grout caused the formation of a film [14] as water evaporates and this acts as a binder. This polymer film interconnects all the cement particles and fillers [15]. Moreover, this is more suitable for household tiling than industrial or special type tiling and the low price for a high outcome is the pivotal reason for its popularity.

This article will give an introductory idea about the components that are being used in making ceramic tile adhesive, especially the inclusion of RPP.

## 2 Components of RPP modified CTA

**Figure 2:**
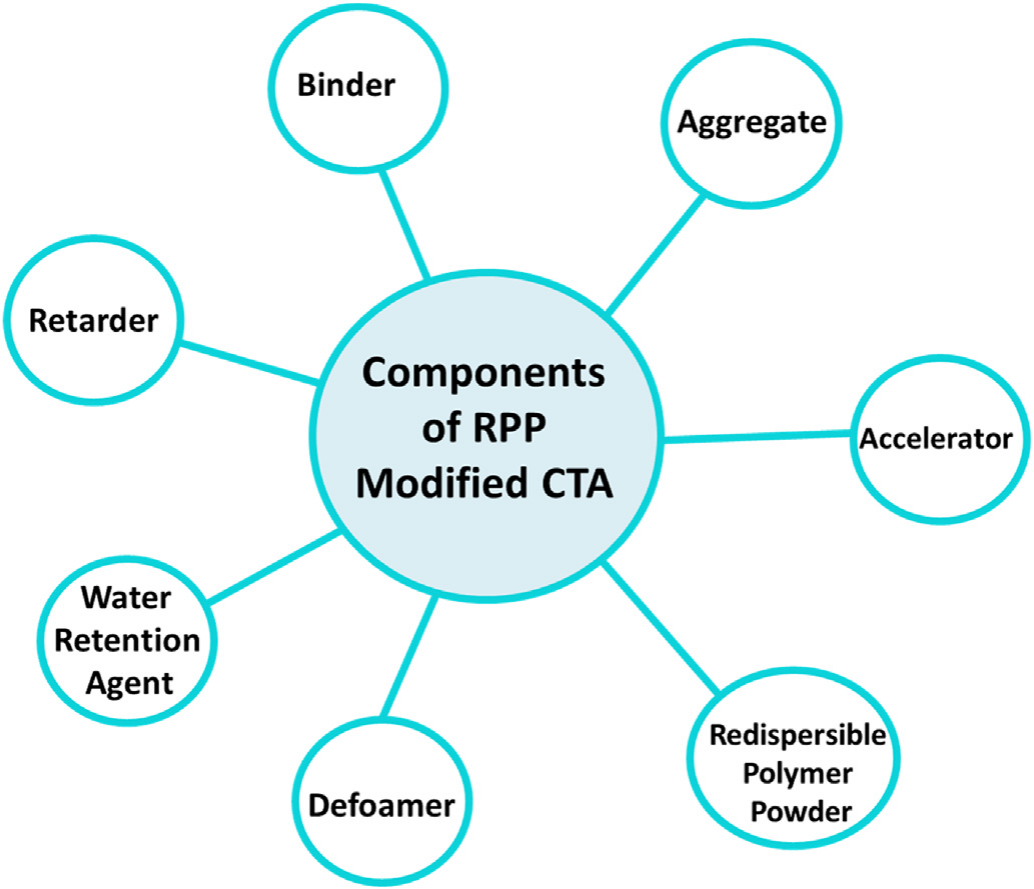
Different components of RPP modified CTA.

CTA has got the exploitation as thin bed mortar for the cladding of tiles in both horizontal and vertical surfaces. A number of components can be added to cementitious tile adhesive (Figure 2) as admixture to impart various properties or to improve existing properties.

### 2.1 Binder

Binder, as the name suggests, holds all the other materials together by cohesion and it does form a bond between the background and the adherent. Cement is the most widely used binder used in construction. Cement, being a powdered material, exerts adhesion by unifying different fragment of solids and converting them into a whole mass [16]. Apart from its use in making concretes, it had been applied for making ceramic tile adhesive along with other admixtures. Different types of cement are existent due to various compositions of the constituents [17]. Three types of cement have been extensively used in making tile adhesive: Portland cement (PC), portland pozzalan cement (PPC) and high-alumina cement (HAC) [8].

Portlant cement is the most prevalent among all the cement types [18] and finds its application as a binding material in ceramic tile adhesive quite easily. Many researchers had been using portland cement as the binding agent for the formulation of tile adhesive [3, 7, 19, 20, 21, 22, 23, 24].

### 2.2 Aggregates

Aggregates are generally used to impart packing density, flexural strength and durability. The composition, size and shape of the aggregates cause an impact on the total mass. So, for making a decent mix, aggregates must be clean, free of clay and other chemicals and also they should not react with other cement constituents. The prevailing aggregates are sand, quartz, limestone, gravel, crushed stone etc. According to Dr. Felixberger [8], silica sand has been the profusely exploited aggregate, mostly used for thin bed tile adhesive with the size range of 0.05mm–0.5mm [8]. Recycled aggregates (RA) could also be an alternative source of aggregates as such recycling imparts environmental preservation. Compared with natural aggregates (NA), it has been found that RA has higher porosity and thus raises the total porosity of the concrete. Thus, inclusion of RA into concrete also imparts significant mechanical and physical advantages. The size of the aggregate plays a vital role for imparting resistance to chemical attacks. It has been suggested that nano-sized aggregates give better impermeability to chemical attacks, i.e. chloride diffusivity and hence escalates the durability of the concrete grout [25].

### 2.3 Accelerators

Accelerators are added to the grout or paste to expedite the setting time and hardening. Both organic and inorganic compounds have been employed for this purpose. Organic accelerators include propionate, diethanolamine, triethanolamine, urea, glyoxal and formate whereas inorganic accelerators are mainly chlorides, fluorides, silicates, aluminates, borates, nitrites etc. Among these compounds, calcium chloride is the most widely used and most effective accelerator which has a well-known evidence of acceleration [12].

### 2.4 Retarders

The pivotal function of retarders is to delay the cement hydration and provide enough time for the paste or grout to be out in the open [8]. Oxides of Pb and Zn, phosphates, magnesium salts, fluorates and borates are the common inorganic retarders. Na, Ca and NH_4_ salts of lingnosulfonic acids, adipic acid, citric acid, tartaric acid, gluconic acid, heptonic acid, succinic acid and carbohydrates are the examples of organic retarders. Retardation exerted by sugars was investigated extensively and the main result includes various theories such as precipitation, adsorption, complexation and nucleation [26]. Non-reducing sugars were found to be more effective retarder than reducing sugars based on the fact that which sugar increases the amount of silica in the solution. Citric acid provides acceleration in the initial stages along with retardation in later stages by forming a complex with monosulfates. The hydration of tricalcium-aluminate (C_3_A) and tricalcium-silicate (C_3_S) is retarded by lignosulfonates and both commercial as well as sugar free lignosulfonates showed excellent results of retardation. Among the inorganic retarders, ZnO doesn't affect the hydration of C_3_A and gypsum but retards the hydration of C_3_S. The presence of Ca(OH)_2_ was not detected because of the formation of calcium hydroxyzincate as it reacts with ZnO [12].

### 2.5 Water retention agents

Water is added to the cementitious mixture in order to hydrate the cement in the first place. When water is evaporated, further water is added to complete the hydration of cement. But, if water retention agents are used, then they can retain the requisite amount of water for complete hydration and enhance the adhesive's non-slump property. The incorporation of water retention agents has become a necessity as tile adhesive bed has transformed from thick bed to thin bed causing rapid dehydration [8]. Cellulose ethers are the most commonly used water retention agents which also help the polymeric powder for better adhesion. Among different types of cellulose ethers, four types are the most widely used; Methyl cellulose (MC), hydroxylpropyl methyl cellulose (HPMC), hydroxyethylmethyl cellulose (HEMC) and hydroxyethyl cellulose (HEC) [27]. Compared to methyl cellulose or hydroxylpropyl methyl cellulose, hydroxyethylmethyl cellulose shows higher solubility, less air entrainment and higher water retention. These are the reasons for picking hydroxyethylmethyl cellulose (HEMC) over others in tile adhesive.

### 2.6 Anti-foaming agents/defoamers

When the admixtures along with cement are mixed with water by means of stirring, it leads to the formation of foam but trapping the air within. To prevent this occurrence, several chemical compounds are used, denoted as anti-foaming or defoaming agents. Commonly used defoamers include insoluble oils, polydimethylsiloxanes placed on a silica carrier, certain alcohols, polyalkylene glycols, stearates etc. [8]. J Xing et. al. investigated the influence of four different types of anti-foaming agents, i.e. mineral oil, polyether, emulsified silicone oil and Polyether modified silicone, on concrete. Experimental data concluded polyether modified silicone to be the best anti-foaming agent among these [28].

### 2.7 Redispersible polymer powders

**Figure 3:**
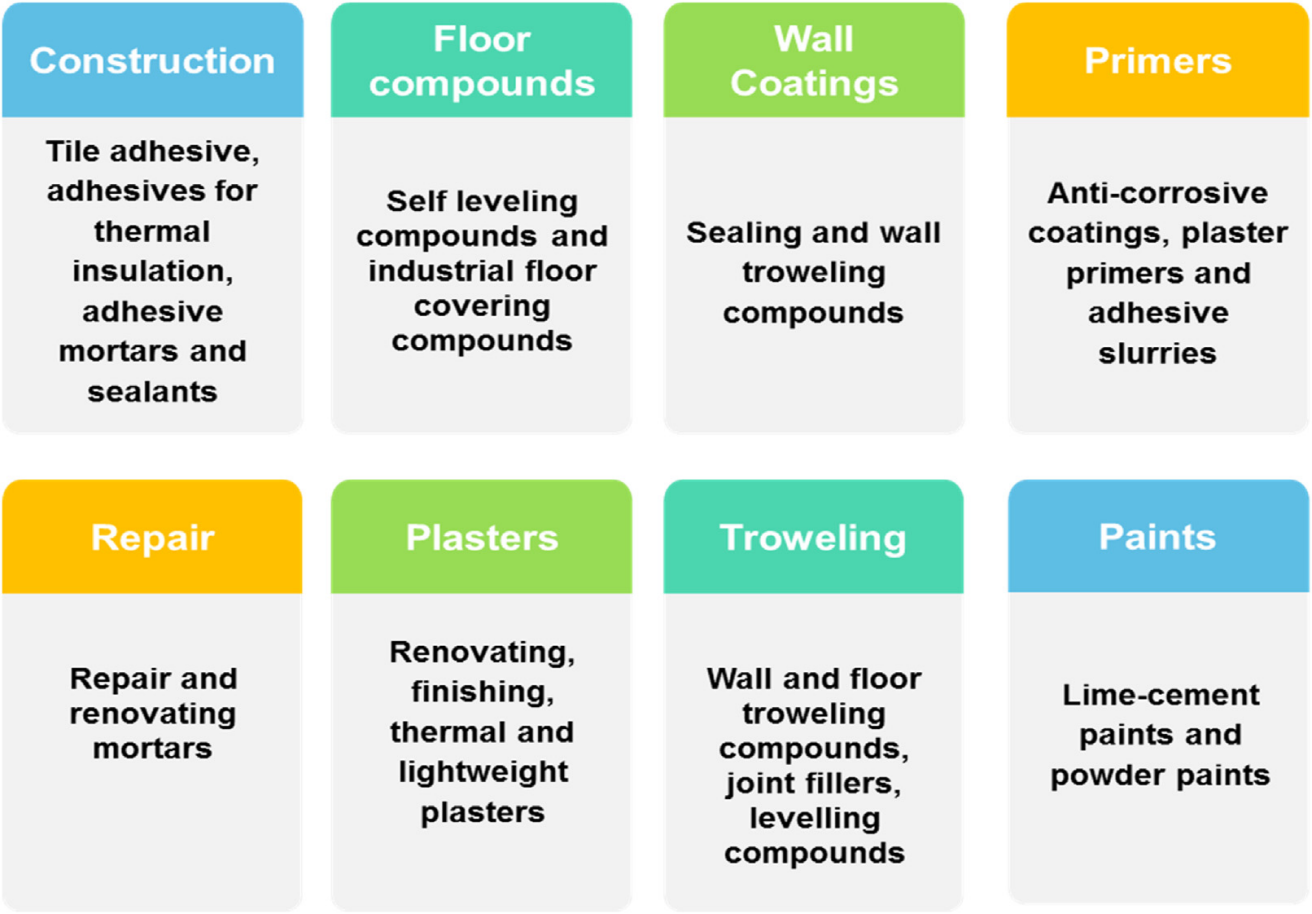
Various applications of RPP.

Since its invention by Wacker Chemie in 1953, RPPs had been exerting a significant impact in modern tile cladding technology [29]. RPP has the ability to impart certain advantageous properties to the cement grout such as: (a) improvement of the tensile strength, plasticity, abrasion resistance and flexural strength of the grout, (b) makes the cement mortar to have certain flexibility by reducing the elastic modulus depending on the cement-polymer ratio, (c) the polymeric film closes the pores and crevices of the hardened grout which makes it impermeable to certain fluids like water, alkali etc. (d) improves the liquidity and constructability of the grout, (e) imparts the water retention property of the grout, (f) provides slip and impact resistance which aids in preventing the formation of cracks. RPP is also employed in different aspects (Figure 3) apart from tile adhesive formulation [15, 30].

**Figure 4:**
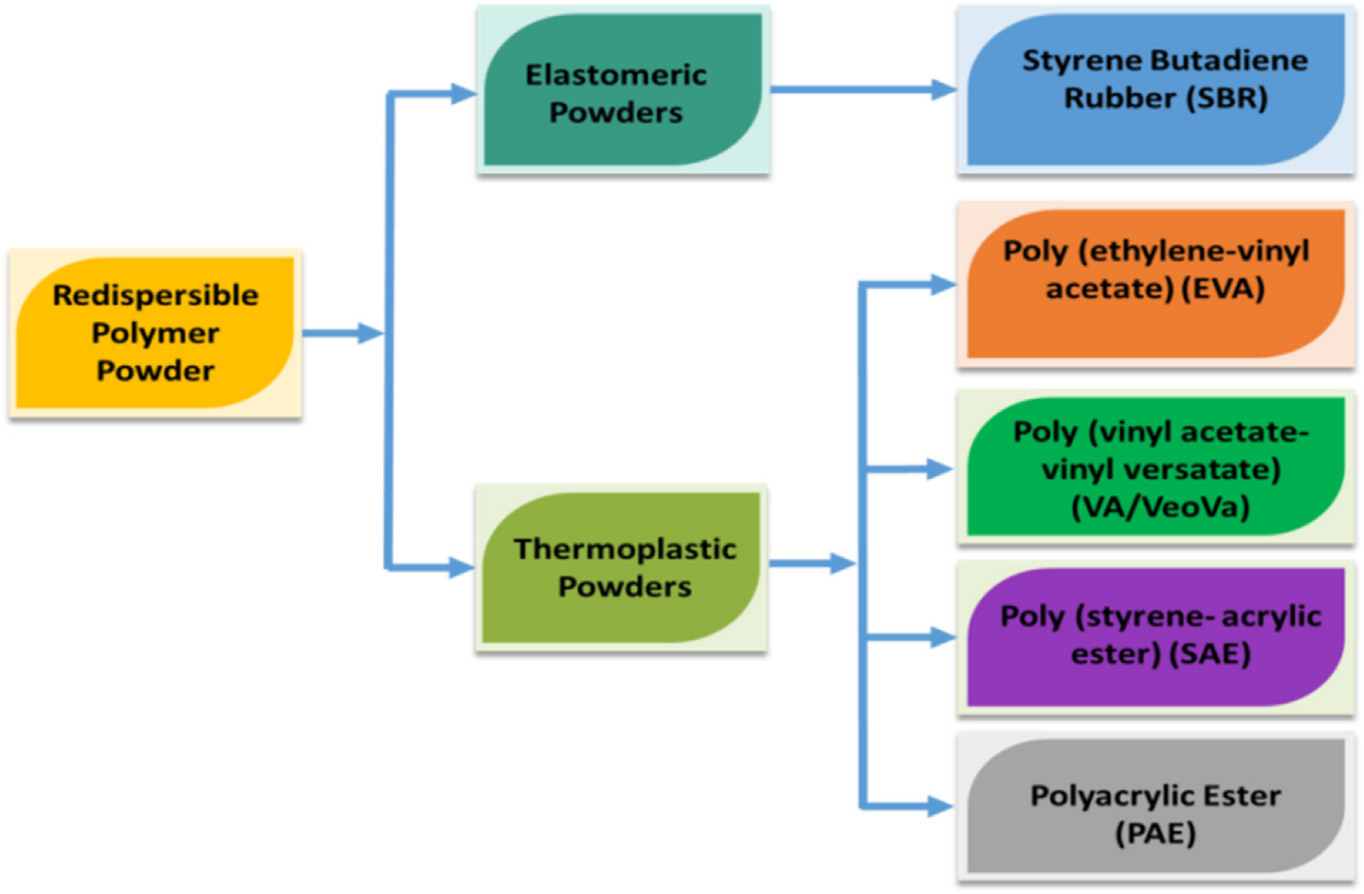
Classification of commercially available RPP.

Commercially available RPP can be classified into two types (Figure 4): the first one is elastomeric powders and the second one is thermoplastic powders. Elastomeric powders include styrene butadiene rubber (SBR) whereas thermoplastic powder includes poly (ethylene-vinyl acetate) (EVA), poly (vinyl acetate-vinyl versatate) (VA/VeoVa), poly (styrene - acrylic ester) (SAE), polyacrylic ester (PAE) [30]. Table 1 provides the acronyms and chemical structures of commercially available RPPs.

**Table 1:** Chemical structures and acronyms of commercially available RPP [12].

Polymer type	Abbreviation	Chemical structure
Styrene Butadiene Rubber	SBR	
Poly (ethylene-vinyl acetate)	EVA	
Poly (vinyl acetate- vinyl versatate)	VA/VeoVa	**-**
Poly (styrene- acrylic ester)	SAE	
Polyacrylic Ester	PAE	

Styrene Butadiene Rubber (SBR) is a wide distribution polymer which initially was used in tire manufacturing. SBR found its first industrial application during World War II as a substitute of natural rubber [31]. Later, it was introduced in concrete industry as a polymer modifier [32] and had been used since [33, 34]. As, the structure of SBR consisted of flexible butadiene and rigid styrene chain, it has shown improved adhesion, durability, mechanical properties, water tightness etc in concrete mortars [35, 36, 37, 38]. Poly (ethylene-vinyl acetate) or EVA is also one of the widely used RPP. Due to its excellent compatibility with cement based system, it has been one of the standard choices in dry-mix mortars [23, 39, 40]. Poly (vinyl acetate-vinyl versatate) or VA/VeoVa is also another RPP under the scrutiny of researchers. The existence of versatate group introduces three long α-alkyl molecule side chains into the polymer that brings extraordinary property like superior alkali resistance [20, 21, 41, 42]. Poly (styrene - acrylic ester) or SAE is a member of the acrylic polymer and has been used to modify cement mortars. With the increase of SAE/cement ratio, the water-reduction and water retention effect, compressive strength, flexural strength and water proofing increases [43, 44, 45, 46]. Appreciable workability and improved mechanical properties were also found for polyacrylic ester or PAE which is also used in making modified mortars [47, 48, 49, 50, 51, 52].

## 3 Process technology of RPP production

The process technology for the production of RPP includes the implementation of a spray drier which atomizes the polymer solution. The hot air converts them into fine particles of 5–500μm size which when placed in water under agitation, disperses having particle size of 1–10μm. The polymer solution is prepared by emulsion polymerization process which includes monomers like olefins and unsaturated monomers of vinyl esters as well as acrylic asters. Emulsion polymerization yields polymer solution with 40–60% solid content. Various additives such as defoamers, thickeners etc. are also added onto this polymeric solution. This polymeric solution is then subjected to spray drying in order to convert this solution into free flowing powder. The purpose of converting this solution into powder is to avoid the tackiness and film forming nature of the adhesive at room temperature. Also, the powder form can easily be stored and handled while maintaining its redispersibility.

**Figure 5:**
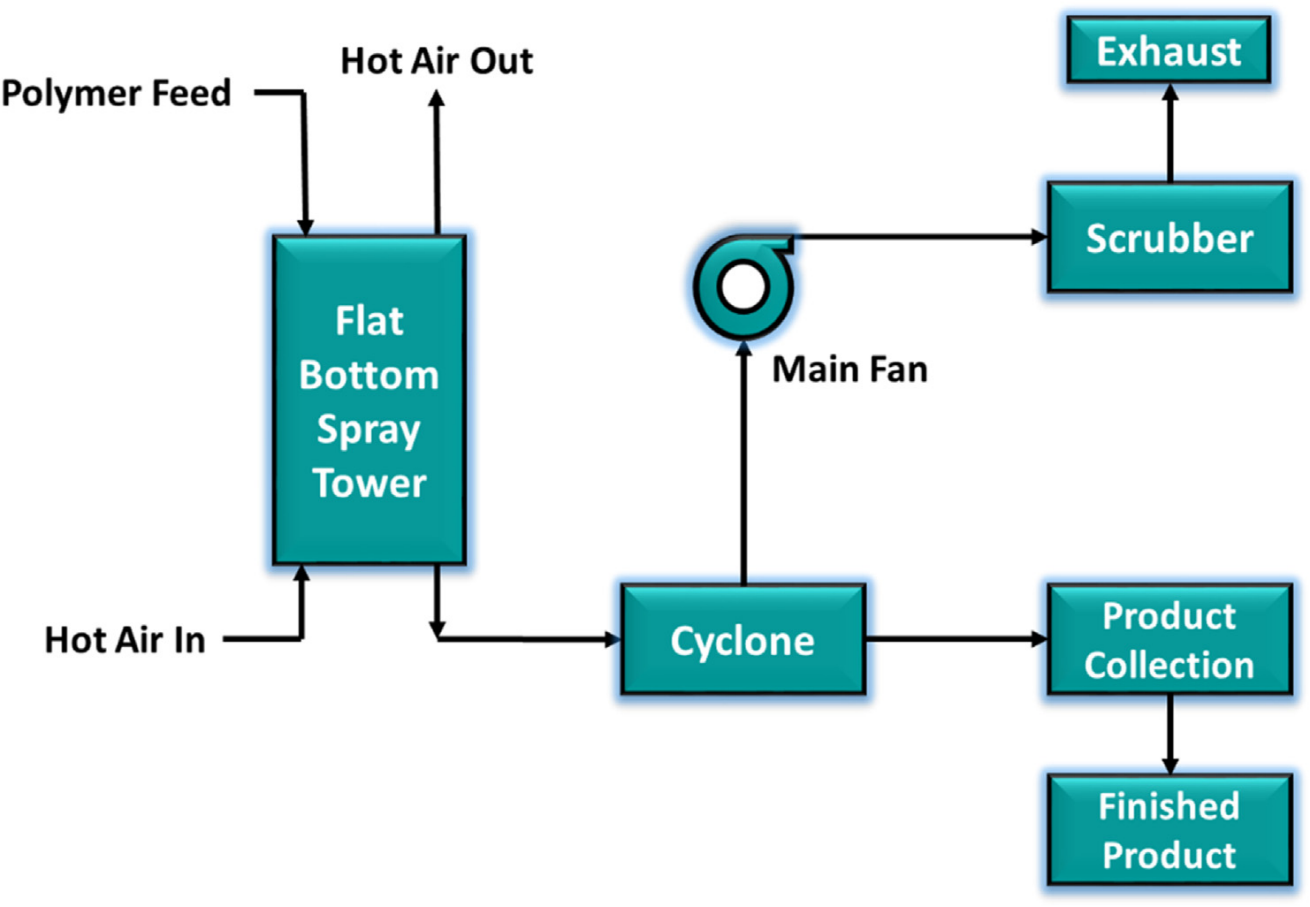
Flow sheet of manufacturing process for RPP.

The spray dryer atomizes the solution into small droplets and a hot air (normal air or nitrogen) flowing counter currently vaporizes the solvents resulting solid powders. Some technologies adopted the con-current flow of droplets and the hot air. Anti-caking agents like CaCO_3_ are added to prevent cake formation of this obtained powder [30]. Flow sheet of manufacturing process for RPP is shown in Figure 5 and typical properties are listed in Table 2.

**Table 2:** Typical properties of RPPs [30].

Type of RPP	Appearance	Average particle size (μm)	Bulk Density (g/cm^3^)	pH (redispersed, 50% solid)
VA/VeoVa	White Powder	10–250	0.54–0.64	4
EVA	White Powder	70	0.40	5–6
PAE	White Powder	45–75	0.31–0.51	10–12
SBR	White Powder	5–50	0.40	7–8

## 4 Typical formulation of a CTA

There are two classes of tile adhesives affixed in the European standard; (a) C1 class and (b) C2 class [53]. C1 class has the limitation of not being able to be used for the fully vitrified tiles and also in places where thermal stress is expected to be higher (balconies, rooftops, terraces etc.). On the other hand, C2 class adhesive has the advantage of being able to be used for all types of tiles and substrates. The main differentiating factor between C1 and C2 class tile adhesive is the quantity of RPP being mixed with other components. The formulation (Table 3) is merely a guideline for the manufacturers which varies upon the type and size of the selected components. Currently a minimum of 15 types of components are being used for the cementitious tile adhesive [8].

**Table 3:** Typical formulation of a CTA [8].

Component name	Example	Dosage (%)	
		C1 class	C2 class
Binder	Portland cement	30–50	30–40
Aggregates	Silica sand	45–70	45–60
Co-filler	Calcium Carbonate	5–10	5–10
Redispersible polymer powder	SBR, EVA, VA/VeoVa, SAE, PAE	0–3	3–6
Water Retention Agents	HEMC	0.2–0.5	0.2–0.5
Accelerator	Calcium formate	<1	<1

## 5 Effect of RPP addition on hydration of CTA

One of the most advantageous features of RPP modification is its redispersion property. When mixed with water, the polymeric dispersion acts as a two phase system with solid polymer particles in water. The binder, i.e. cement present in the CTA becomes a paste and the RPP particles get uniformly dispersed in it. This results in improved toughness and durability of the system. Chandra and Flodin [54] proposed two theories for the action of polymer dispersion during cement hydration present in CTA mortar. According to the first theory, no chemical reaction occurs between RPP and cement constituents. The available water gets depleted as it is being used in cement hydration. This results in coalescence of RPP which begets the gradual formation of a three dimensional polymeric network that serves in bolstering the strength and toughness of the CTA mortar [55, 56]. According to the second theory, not only this phenomenon happens but also chemical interaction occurs between RPP and cement hydration products. This results in formation of complexes that delay or hasten the hydration of the cement system [54, 57, 58].

## 6 Methods to evaluate RPP modified CTA

### 6.1 Adhesion strength

The use of RPP on cement-sand grout or mortar can be assessed by a most commonly used mechanical technique known as adhesion strength or bond strength or tensile strength or tensile adhesion test. Adhesion strength refers to the maximum strength per unit surface area which can be measured by shear (EN 1324:2007, EN 12003:1997) or tensile strength (EN 1348:2007) [59, 60, 61]. This test of adhesion strength follows the European standard DIN EN 12004:2007 resp. ISO 13007-1:2006 [53, 62].

Schulze [11] carried out an investigation regarding the adhesion strength of RPP in mortars for a time period of 10 years. The effect of modification with EVA (Poly (Ethylene-Vinyl Acetate)) and SAE (Poly (Styrene- Acrylic Ester)) in cement (CEM 1 32.5R) and sand mixture was evaluated, compared with a blank sample which included only cement and sand mixture. The exploration was carried out in indoor and outdoor climate exposure. In case of outdoor climate condition, initially (after 28 days) the adhesion strength of EVA modified mortar was higher than SAE modified mortar which was higher than normal cement-sand mixture. Gradual increase in the adhesion strength was observed for all the grouts. The maximum bond strength was found after 10 years. At the end of 10 years, the blank sample (ordinary cement-sand mixture) gained the adhesion strength which was even less than the EVA modified mortar's initial value. In indoor climate condition, the blank or unmodified mortar showed no increase and stayed below 0.5 N/mm^2^. EVA and SAE performed similarly and showed slight increase in adhesion strength at a time spent of 10 years. The fizzle of blank sample or unmodified mortar to impart strength even in a longer time period might be due to the inability of the mortar to retain adequate amount of water for the setting of cement. The modification with a EVA and SAE powder acted as the binder to hold the aggregates together which resulted in better adhesion.

J.Y petit [19] conducted an experiment to figure out the correlation between formulation parameter and tile adhesive property. The RPPs chosen for the test was VA/VeoVa and EVA powder. CTA mortars of viscosities 76,000 and 70,000 mPa.s respectively were prepared by adding them. The formulation included cement and sand mixture with 2 wt% RPP content. The bond strength was measured at 28 days on a 20 minute open time, based on which the mortar formed with VA/VeoVa powder showed decent result. The bond strength was found out to be three times higher than that of the EVA powder. The reason might be the depletion of water by evaporation that pioneered the polymer film formation. This resulted in better mechanical anchoring of C–S–H gel from cement's hydration into the pores of the tiles.

### 6.2 Flexural strength

Flexural strength is defined by the extent to which an object may resist breakage when bent [63]. It is the measure of maximum amount of load which a specimen can bear before permanent deformation. The flexural strength of cementitious tile adhesive is determined by following the standard EN 12808-3:2002 [64].

The inclusion of polymeric resin or RPP into the cement-sand mixture, evidently increased the flexural strength in the early ages [65, 66]. This was because of the fact that the polymeric material gets engrossed into the pore system and does the reinforcement. The flexural strength of the blank sample or the unmodified mortar increased twice as much value to its initial (28 days) value after one year in outdoor climate condition. EVA modified mortar performed less in flexural strength than the SAE modified mortar in the outdoor climate condition. This might be resulting from the degree of hydration of cement. The blank sample got the maximum value of flexural strength after 10 years in indoor climate condition which is only tantamount to the value after 1 year in outdoor climate condition. Presence of low water content in indoor condition contributed to the lower degree of hydration which ultimately lowers the flexural strength.

From the research work carried out by Afridi [66], VA/VeoVA, EVA and SBR RPP were incorporated in the mortar. The flexural strength evidently increased by this inclusion. According to the work of Barluenga et. al. [67], the flexural strength of LMM (latex modified mortar) increased with the increased percentage of latex.

### 6.3 Compressive strength

The compressive strength may be defined by the ability of a structure to withstand maximum amount of load on its surface until it faces any deflection or crack. The evaluation of compressive strength is a very monumental investigation for the study of concrete as it provides valuable information regarding the performance during service conditions. According to EN 13888:2002, compressive strength is defined by the maximum value of a grout prism failure determined by exerting a force in compression on two opposite points [68]. The European standard EN 12808-3:2002 [64] is followed for the evaluation of compressive strength.

The effect of RPP inclusion on the compressive strength of the cement-sand mixture was investigated by Afridi [66]. In his literature, the compressive strength of the grout increased for both the powdered and aqueous polymer modified mortar. This was attributed as the water-cement ratio got decreased. The capillary porosity of the system also got reduced with the decreasing water-cement ratio. As a result, finer porosity of the system is obtained as the pore size distribution shifted towards the finer porosity. The polymeric film that formed on the grout increased the compressive strength but in a lesser extent. In case of VA/VeoVA modified mortars, the compressive strength reduced in minute amount because of its high air content. Table 4 lists the influence of RPP modification on the mechancal properties (adhesion, flexural and compressive stength) of grout.

**Table 4:** Influence of RPP modification on the mechanical properties of grout.

RPP	Mix Design	P:C	Mechanical Properties			Ref.
			Adhesion Strength	Flexural Strength	Compressive Strength	
SBR	PC, S & W	0.176	7.5 MPa	12.2 MPa	-	[69]
	OPC, S, SP & W	0.176	-	8.9 MPa	49.1 MPa	[52]
	OPC, S & W	0.07	21 Kgf cm^−2^	-	-	[70]
	PC, S & W	0.015	-	9 MPa	64 MPa	[38]
	OPC, S, CA, W, SP & AFA	0.25	-	11.5 MPa	-	[71]
	OPC, S, CA, FA & W	0.176	-	3715 psi		[35]
	PC, S & W	0.25	-	106 Kgf cm^−2^	340 Kgf cm^−2^	[66]
EVA	PC, S & W	0.13	2.55 N/mm^2^	12.7 N/mm^2^	43 N/mm^2^	[11]
	OPC, S & W	0.03	-	-	52 N/mm^2^	[14]
	OPC, S & W	0.07	22 Kgf cm^−2^	-	-	[70]
	OPC, S, DF, SP & W	0.02	-	-	67 N/mm^2^	[72]
	OPC, S, CA & W	0.19	-	3.7 MPa	23.3 MPa	[39]
	RHSAC, FOA, WRA, SP & W	0.1	-	-	250 kPa	[73]
	PC, FA, AEA, AFA & W	0.086	21.33 MPa	-	28.90 MPa	[74]
	OPC, S, SP & W	0.176	-	7.7 MPa	36.7 MPa	[52]
	OPC, S & W	0.25		120 Kgf cm^−2^	337 Kgf cm^−2^	[66]
VA/VeoVa	OPC, S & W	0.02	12 Kgf cm^−2^	-	-	[70]
	PC, S & W	0.15	-	10 MPa	-	[41]
	PC, FA, AEA, AFA & W	0.086	29.02 MPa	-	43.20 MPa	[74]
	OPC, S & W	0.25	-	105 Kgf cm^−2^	275 Kgf cm^−2^	[66]
SAE	PC, S & W	0.13	2.47 N/mm^2^	14.5 N/mm^2^	55 N/mm^2^	[11]
	OPC, S & W	0.06	2194 N	-	27 N/mm^2^	[44]
	PC, S & W	0.176	6.6 MPa	18.7 MPa	-	[69]
PAE	OPC, S, SF, SP & W	0.43	5.27 MPa	-	-	[47]
	OPC, S, SP & W	0.176	-	7.5 MPa	35.8 MPa	[52]

∗∗∗RPP = Redispersible Polymer Powder, P:C=Polymer:Cement Ratio, PC = Portland Cement, OPC = Ordinary Portland Cement, S=Sand, W=Water, SP = Super Plasticizer, CA = Coarse Aggregate, FA = Fine Aggregate, AFA = Anti Foam Agent, DF = Defoamer, FOA = Foaming Agent, WRA = Water Retention Agent, AEA = Air Entraining Agent, SF = Silica Fume.

The study conducted by Schulze [11] with EVA and SAE modified mortar; the results showed a reduction in compressive strength. In both indoor and outdoor climate condition, the compressive strength of the blank sample (unmodified mortar) which contained no polymer powder showed the maximum value of compressive strength compared to EVA and SAE modified mortars. The value was highest from the initial 28 days exposure to 10 years, with all the successive years. The RPPs were soft materials compared to the cement-sand aggregates. This led to the reduced compressive strength of the modified mortar. The experiment carried out by Barluenga et. al. [67]. resulted a relatively constant compressive strength for LMM with SBR at 28 days.

### 6.4 Water-retention rate

A decent water-retention rate is very much beneficent for the construction because of the fact that it imparts certain properties of the mortar. This water-retention rate is presented as a quantitative index in order to evaluate the water-retention effect of that mortar [21]. The water-retention rate can be assessed following DIN18555-7 [75].

According to the findings of R. Wang et al. [21], the rate of water-retention were increasing quite significantly with the increase of cement to mortar ratio and continued to do so. In this experiment, VA/VeoVa polymer powder was mixed with cement and the ratio was up to 20%. The sharp raise was evident until it reached 98% of water-retention rate which corresponded to 6% polymer to cement ratio. R. Wang et al. pointed out three seasons behind this excellent water-retention rate of this mortar. The first reason was the good water reduction property of VA/VeoVa polymer powder which reduced the unit water usage. Induction of excellent water distribution by VA/VeoVa polymer powder was the second reason and thirdly, the blocking effect of VA/VeoVa powder on water made its separation from the system very difficult.

### 6.5 Water absorption

The amount of water that is absorbed by the cementitious tile adhesive plays a very important role in the serviceability and performance. The European standard EN 12808-2:2002 is the guideline for water absorption test [76]. Water absorption is generally measured by weighing a dried sample until constant weight, submerging it in water for a certain period of time and then weighing it as a percentage of dried weight [77, 78, 79]. The generalized formula for calculating the percentage of water absorption is,

$$\%\;Water\;Absorption=\;\frac{W_{wet\;}-W_{dry}}{W_{dry}}\;\times 100$$

Here, *W*_*dry*_ = weight of the dry sample.

*W*_*wet*_ = weight of wet sample.

### 6.6 Shrinkage

Shrinkage refers to the reduction in length of the grout. Due to the evaporation of water and chemical changes, shrinkage occurs. EN 12808-4:2002 is the European standard for the measurement of shrinkage [80].

For a mortar modified with VA/VeoVa polymer powder, the shrinkage rate was increasing in a very minute manner for a polymer powder-cement ratio of 6%. Further increase in the powder-cement ratio caused the shrinkage rate to reduce sharply and this reduction continued until the powder-cement ratio reached 20%. This reduction of shrinkage rate of the mortar by VA/VeoVa polymer powder may be due to the formation of polymer film that obstructed the water escape from the system [21]. According to the work of Weng et. al [74], for a water-cement ratio of 0.5, the drying shrinkage rate increased from 0.0128% (reference) to 0.0224% for the addition of 8% EVA powder whereas addition of 8% VA/VeoVa powder increased it to 0.0159% which is slightly less efficient than EVA addition. Better results were achieved when the water-cement ratio was increased to 0.6 and like before, EVA addition showed better rate than the VA/VeoVa addition.

## 7 Challenges and future research & developments of CTA

Like any other technological venture, CTA faces many challenges. Out of these challenges, the prevalent one is lessening the failure of tiling system. Ceramic tiles, when installed in outdoor climatic conditions, faced a wide variety of damages [2]. Chew [81] enlisted a number of reasons that causes the failure of tiling system; a) the deformation of mortar comprising CTA onto which ceramic tiles laid due to shrinkage, b) due to thermal, moisture or other effects, differential movement is induced between ceramic tiles-CTA-substrate which leads to failure, c) failure of the cement rendering behind the adhesive, d) inappropriate surface preparation such as inadequate cleaning, no dispensation of proper keys etc. e) structural movements like vibrations and settlement problems, f) inopportune selection and scheme of materials. To eradicate such failures, Wetzel et al. [82] suggested some points which can be adopted during the tiling system; a) appropriate choice of materials considering their size and workability, b) compatible structural design e.g. water drainage, flexible waterproofing, c) congenial installation practices (e.g. pretreatment of substrate and tiles). To investigate the effect of weathering conditions, Yiu et al. [83] did the research on wind, rain, moisture and pollutants-attack on external tiling systems. This was the first laboratory based investigation done on this regard. Significant results were obtained as it showed 50% decrease of shear strength for the first 100 cycles and this can't be ignored.

According to a market survey [84], in 2018 the global CTA market was valued at USD 15.08 billion and is projected to reach USD 40.73 billion by the year 2026. Although it is very challenging to quantify the size of CTA market, it is indisputable that CTA's are one of the significantly growing building materials on current market [85]. In order to continue CTA's upward trend in the market, further research and development is an indisputable aspect. In addition, assessment programs like Polish Market Surveillance Authorities can be carried out to assure the consistency of the performance of CTA [86].

## 8 Conclusion

The drawbacks of using ordinary cement-sand grout or paste can easily be eradicated by the inclusion of RPP along with other constituents which comprises the CTA for ceramic tiles. This surely provides better adhesion, better flexural strength, better water retention, better resistance to chemical attack, improved abrasion resistance and many other quality aspects. Formulation of CTA is economically feasible because the quantity of polymer powder is required in lesser amount. The easy preparation and application of this tile adhesive makes it suitable for household tiling purposes. Due to such flexibility, CTA has a great future.

## Declarations

### Author contribution statement

All authors listed have significantly contributed to the development and the writing of this article.

### Funding statement

This research did not receive any specific grant from funding agencies in the public, commercial, or not-for-profit sectors.

### Data availability statement

Data will be made available on request.

### Declaration of interests statement

The authors declare no conflict of interest.

### Additional information

No additional information is available for this paper.
